# Identification of the shared gene signatures between pulmonary fibrosis and pulmonary hypertension using bioinformatics analysis

**DOI:** 10.3389/fimmu.2023.1197752

**Published:** 2023-09-04

**Authors:** Hui Zhao, Lan Wang, Yi Yan, Qin-Hua Zhao, Jing He, Rong Jiang, Ci-Jun Luo, Hong-Ling Qiu, Yu-Qing Miao, Su-Gang Gong, Ping Yuan, Wen-Hui Wu

**Affiliations:** ^1^ Department of Cardio-Pulmonary Circulation, Shanghai Pulmonary Hospital, School of Medicine, Tongji University, Shanghai, China; ^2^ School of Materials and Chemistry & Institute of Bismuth and Rhenium, University of Shanghai for Science and Technology, Shanghai, China; ^3^ Heart Center and Shanghai Institute of Pediatric Congenital Heart Disease, Shanghai Children’s Medical Center, School of Medicine, Shanghai Jiao Tong University, Shanghai, China

**Keywords:** pulmonary fibrosis, pulmonary hypertension, WGCNA, differential gene analysis, T cells CD4

## Abstract

Pulmonary fibrosis (PF) and pulmonary hypertension (PH) have common pathophysiological features, such as the significant remodeling of pulmonary parenchyma and vascular wall. There is no effective specific drug in clinical treatment for these two diseases, resulting in a worse prognosis and higher mortality. This study aimed to screen the common key genes and immune characteristics of PF and PH by means of bioinformatics to find new common therapeutic targets. Expression profiles are downloaded from the Gene Expression Database. Weighted gene co-expression network analysis is used to identify the co-expression modules related to PF and PH. We used the ClueGO software to enrich and analyze the common genes in PF and PH and obtained the protein–protein interaction (PPI) network. Then, the differential genes were screened out in another cohort of PF and PH, and the shared genes were crossed. Finally, RT-PCR verification and immune infiltration analysis were performed on the intersection genes. In the result, the positive correlation module with the highest correlation between PF and PH was determined, and it was found that lymphocyte activation is a common feature of the pathophysiology of PF and PH. Eight common characteristic genes (*ACTR2, COL5A2, COL6A3, CYSLTR1, IGF1, RSPO3, SCARNA17* and *SEL1L*) were gained. Immune infiltration showed that compared with the control group, resting CD4 memory T cells were upregulated in PF and PH. Combining the results of crossing characteristic genes in ImmPort database and RT-PCR, the important gene *IGF1* was obtained. Knocking down *IGF1* could significantly reduce the proliferation and apoptosis resistance in pulmonary microvascular endothelial cells, pulmonary smooth muscle cells, and fibroblasts induced by hypoxia, platelet-derived growth factor-BB (PDGF-BB), and transforming growth factor-β1 (TGF-β1), respectively. Our work identified the common biomarkers of PF and PH and provided a new candidate gene for the potential therapeutic targets of PF and PH in the future.

## 1 Introduction

Pulmonary hypertension (PH), defined as a mean pulmonary artery pressure (mPAP) >20 mmHg at rest (1), is a major global health issue, with an estimated prevalence of ~1% in the population worldwide and 10% in individuals aged >65 years (1). PH is often accompanied by pulmonary fibrosis (PF). In idiopathic pulmonary fibrosis (IPF), 8%–15% of patients have PH at diagnosis, and the prevalence is raised to 30%–50% in the advanced stage and >60% at the end stage of the disease. PH significantly complicates the course of PF and heralds an unfavorable disease prognosis (2, 3). As a well-known fatal lung disease, the average life expectancy of IPF is 3–5 years after diagnosis (4, 5), and the mortality climbs even higher when pulmonary vascular remodeling is involved (6–8). Currently, although several drugs targeting group 1 PH [pulmonary arterial hypertension (PAH)] have been studied in patients with PF associated with PH, the results are inconsistent, with some studies indicative of ineffective or even harmful impact (9–11), which unfortunately left lung transplantation the only durable treatment for PH secondary to PF (6). Therefore, finding a new common therapeutic target for PF-PH is particularly important.

The pathological feature of PF associated with PH involves an overexuberant fibroproliferative process gradually destructing the normal lung architecture and sustained remodeling in the pulmonary vasculature. Even though the underlying pathophysiology is complex and unique, accumulating evidence indicates that the development of fibrosis and vascular obliteration (both in PAH and secondary PH) share several similar mechanisms involving epigenetics, metabolism, inflammation, immunity, DNA damage, and oxidative stress (6, 12–14). We have recently reported that targeting checkpoint kinases 1/2 exhibited a promising antifibrotic and antiproliferative activity in PF and pulmonary vascular remodeling (13, 14). However, the pathological origins of PF and PH remain undetermined, and understanding at the systems level and developing novel therapeutic strategies remain highly recommended.

With rapid advances in sequencing technology (15), it is available to measure the expression of thousands of genes in various diseases, which helps people understand the pathogenesis of diseases in depth from the gene level (16). In this study, we identified co-expression modules and shared genes of PF and PH by using publicly available gene expression data from the NCBI Gene Expression Omnibus (GEO) database. The results revealed that PF and PH shared 313 genes in discovery cohorts, and they were mainly enriched in lymphocyte activation. We further validated that *IGF1* was particularly noteworthy, which may serve as the key modulator and potential therapeutic target in PF and PH in the future.

## 2 Materials and methods

### 2.1 Patients and samples

The lung tissues from six PF-PH and five healthy subjects were obtained from patients undergoing an open lung biopsy or lung transplant in Shanghai Pulmonary Hospital in the period from 2020 to 2022. The diagnosis of PF-PH was established from the European Society of Cardiology and the European Respiratory Society guidelines (1). All of the experimental procedures using human tissues were approved and supervised by the Ethics Committee of Shanghai Pulmonary Hospital (numbers: K22-137Y). Written informed consent was obtained from all participants.

### 2.2 Data collection and processing

We used “pulmonary arterial hypertension” or “idiopathic pulmonary fibrosis,” the two typical diseases for pulmonary vascular remodeling and PF, as keywords to search in the GEO database (https://www.ncbi.nlm.nih.gov/geo/) and obtain the expression profiles. Database inclusion criteria were as follows. First, the sequencing methods of the selected database are expression profiling by array, and the dataset must contain both control and disease groups. Second, the tissue sources used for sequencing were all human lung tissue. Third, the number of samples should be sufficient to ensure the accuracy of each analysis, and the database must provide either processed or raw data that can be used for reanalysis. Finally, we selected GSE53845, GSE113439, GSE110147, and GSE15197 from the GEO database. Subsequently, we used R 4.2.0 software to convert probes in GSE53845 and GSE113439 into gene symbols. We provide all involved R programs in **Supplementary Table 1**.

### 2.3 Weighted gene co-expression network analysis

PF- and PH-related modules were gained by weighted gene co-expression network analysis (WGCNA) (17), and WGCNA was carried out using SangerBox (sangerbox.com). The gene cluster map and the correlation heat map between modules and phenotypes were obtained.

### 2.4 Identification of shared unique genes in PF and PH

We selected the modules highly correlated with PF and PH and screened the overlapping shared genes in the modules that are positively associated with PF and PH. ClueGO (18) explored the potential role of these shared genes in PF and PH and made a biological analysis of these shared genes. A p-value <0.05 was considered significant. Protein–protein interaction (PPI) network analysis used STRING (string-db.org), and the “MCODE” algorithm in Cytoscape software was used to realize visualization.

### 2.5 Validation of shared genes through differential expression analysis

To further identify potential functional genes for PF and PH, differential expression analysis was performed using GEO2R between patients and healthy controls in GSE110147 or GSE15197. The screening threshold of differentially expressed genes (DEGs) was set at a log2 |fold change (FC)| >1 and a p-value <0.05. Overlapping DEGs in PF and PH databases were acquired by Venn. A set of immunity-related genes was achieved from the ImmPort database (19) and crossed with identified common genes by Venn mapping.

### 2.6 Immune infiltration analysis and correlation analysis between immune cells and feature genes

The normalized GSE53845 and GSE113439 expression data were analyzed by CIBERSORT (https://cibersort.stanford.edu/) to evaluate enrichment for immune invasions and to obtain an immunocyte infiltration matrix. Furthermore, we displayed the percentage of each immunocyte in the samples in a histogram, constructed a heatmap of 22 kinds of immunocytes, and compared the levels of 22 immunocytes between patients and controls using SangerBox. Spearman’s rank correlation analysis was used to analyze the relationship between immunocytes and feature genes. A p-value <0.05 was considered statistically significant.

### 2.7 Quantitative real-time PCR assay

Lung tissue samples were obtained from patients with PF-PH who underwent a lung transplant and healthy donors (controls) and stored at −80°C until use. Total RNA was extracted from the lung tissue using TRIzol reagent. RNA purity was determined using the NanoDrop 2000 spectrophotometer at 260/280 nm (ratio = 1.9–2.1). Then, reverse transcription using the PrimeScript™ RT reagent Kit (TaKaRa, RR0371, Japan) was performed according to the manufacturer’s instructions. RT-PCR was then performed with the SYBR Green-based RT-PCR (TOYOBO, QPK201, Japan). For RT-PCR analysis, the expression level of genes was represented as a fold change using the 2^−△△Ct^ method, and a p-value <0.05 was considered statistically significant. Primer sequences are listed in **Supplementary Ta** **ble S2**.

### 2.8 Cell culture, transfection, and cell function test

Human pulmonary microvascular endothelial cells (PMECs), human pulmonary artery smooth muscle cells (PASMCs), and human fibroblasts were purchased from ScienceCell (Shanghai, China) and cultured in endothelial cell medium, smooth muscle cell medium, and Dulbecco’s modified Eagle’s medium, respectively, and incubated with 5% CO_2_ at 37°C. Hypoxia-induced PMECs were acquired in anoxic incubators with 5% O_2_ at 37°C. PASMCs and fibroblasts were induced to proliferate with 10 ng/mL platelet-derived growth factor-BB (PDGF-BB) (PeproTech, USA) and 5 ng/mL transforming growth factor-β1 (TGF-β1) (PeproTech, USA), respectively.

Cells were transfected with 20 μM siRNA *IGF1* (Genomeditch, Shanghai, China) using Lipo2000 Transfection Reagent (Invitrogen, USA). The control groups were treated with equal concentrations of negative control sequences to eliminate nonspecific effects.

Cell Counting Kit-8 (CCK-8, Dojindo, Japan) was used to measure cell proliferation. Cells were inoculated into a 96-well plate at a density of 3 × 10^3^ cells per well. After 48 h of siRNA *IGF1* treatment under the stimulation of proliferation induction, cells were treated with 10 μL CCK-8 solution at 37°C for 60 min and then tested. The absorbance at 450 nm was then detected, with a relative proliferation level obtained by normalization from five independent experiments.

In this study, 2 × 10^3^ treated cells were planted in each well of a 96-well plate. After 48 h of treatment, cell proliferation was measured by EdU Kit (BeyoClick™, China). Hoechst (blue) labeled the nucleus and EdU (green) labeled the proliferating cells, and these were observed and counted under the fluorescence microscope.

The Annexin V apoptosis kit (Dojindo, Japan) was used to detect the apoptosis of cells. In this study, 2 × 10^5^ cells were planted into each well of a 6-well plate with or without treatment. After 48 h, cells were digested with trypsin, and cells were washed with phosphate buffered saline (PBS) three times. Then, 500 μL of 1× Annexin V binding solution was added to the washed cells to prepare a cell suspension, and 3 μL of Annexin V-FITC and PI were added, respectively. The samples were mixed thoroughly and were left in the dark for 15 min. Finally, the apoptosis was analyzed by flow cytometry.

The cells were analyzed for fluorescence by Calcein-AM/PI staining (Calcein-AM/PI Double Stain Kit, YESEN, China). Cells were seeded in 24-well plates (2 × 10^5^ cells) for 48 h with or without treatment. Dyeing solution was added, and the cells were incubated at 37°C for 30 min. Finally, the cells were analyzed using a fluorescence microscope.

### 2.9 Molecular docking

In order to further verify the ability of protein IGF1 to target drugs, molecular docking was carried out. Firstly, a database was selected in The NCGC Pharmaceutical Collection, which contained 7,929 FDA-approved drugs. The crystal structure of 2OJ9 was downloaded from the PDB database as the receptor protein structure for molecular docking. Secondly, the Discovery Studio LibDock program was used for molecular docking screening, and the top 20 compounds of LibDock score were selected for further study. Subsequently, for the top 20 compounds, the ligands and protein needed for molecular docking were prepared by AutoDock Vina software (http://vina.scripps.edu/), and for the target protein, the crystal structure obtained from the PDB database (https://www.rcsb.org/) needed pretreatment. Finally, the target structure was molecularly docked with the active ingredient structure, and the affinity value of the target structure is the binding ability of the target structure and the active ingredient structure using vina in pyrx software (https://pyrx.sourceforge.io/).

### 2.10 Statistical analysis

SangerBox (sangerbox.com) was used for all statistical analyses. Differences between the two groups were compared using parametric tests for normally distributed variables. All of the statistical differences were p-value <0.05.

## 3 Results

### 3.1 GEO information

Based on the screening criteria mentioned in the method, we selected four GEO datasets numbered GSE53845, GSE110147, GSE113439, and GSE15197. Furthermore, GSE53845 (20) and GSE113439 (21) served as discovery queues for WGCNA. A concise description of these samples was summarized in **Supplementary T** **able S3**. Additionally, GSE110147 and GSE15197 served as validation queues for DEG analysis.

### 3.2 Key modules associated with PF and PH by WGCNA

**Figure 1:**
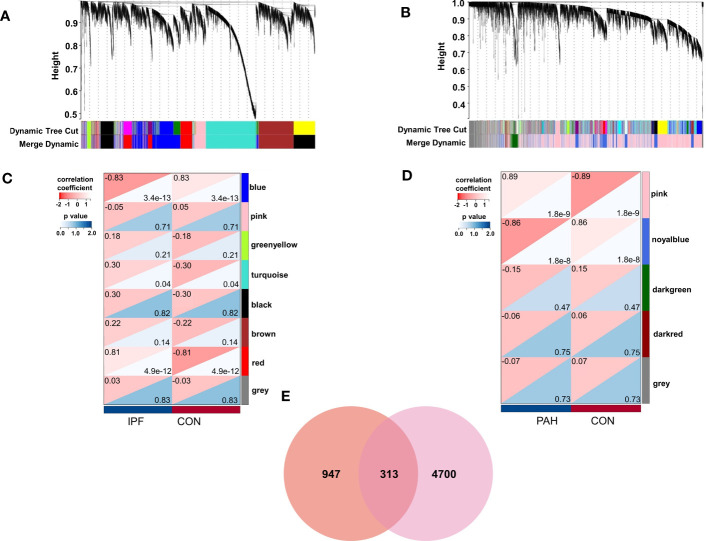
Weighted gene co-expression network analysis (WGCNA). **(A)** The cluster dendrogram of co-expression genes in PF. **(B)** The cluster dendrogram of co-expression genes in PH. **(C)** Module–trait relationships in PF. **(D)** Module–trait relationships in PH. **(E)** Shared genes overlap between the red module of PF and the pink module of PH. IPF, idiopathic pulmonary fibrosis; PAH, pulmonary arterial hypertension; CON, control.

We identified eight relevant modules for GSE53845 through WGCNA, with each color representing a different module. A heat map of the module–trait relationship was then drawn based on the correlation coefficients to assess the association of each module with disease (**Figures 1A, C**). Among them, the “red” module (r = 0.81, p = 4.9e-12) had a high correlation with PF and was selected as the module with the highest positive correlation with PF, including 1,260 genes. Similarly, in GSE113439, module “pink” (r = 0.89, p = 1.8e-9) was the module with the highest positive correlation with PH, including 5,013 genes (**Figures 1B, D**).

### 3.3 Common genes and biological functions in PF and PH

**Figure 2:**
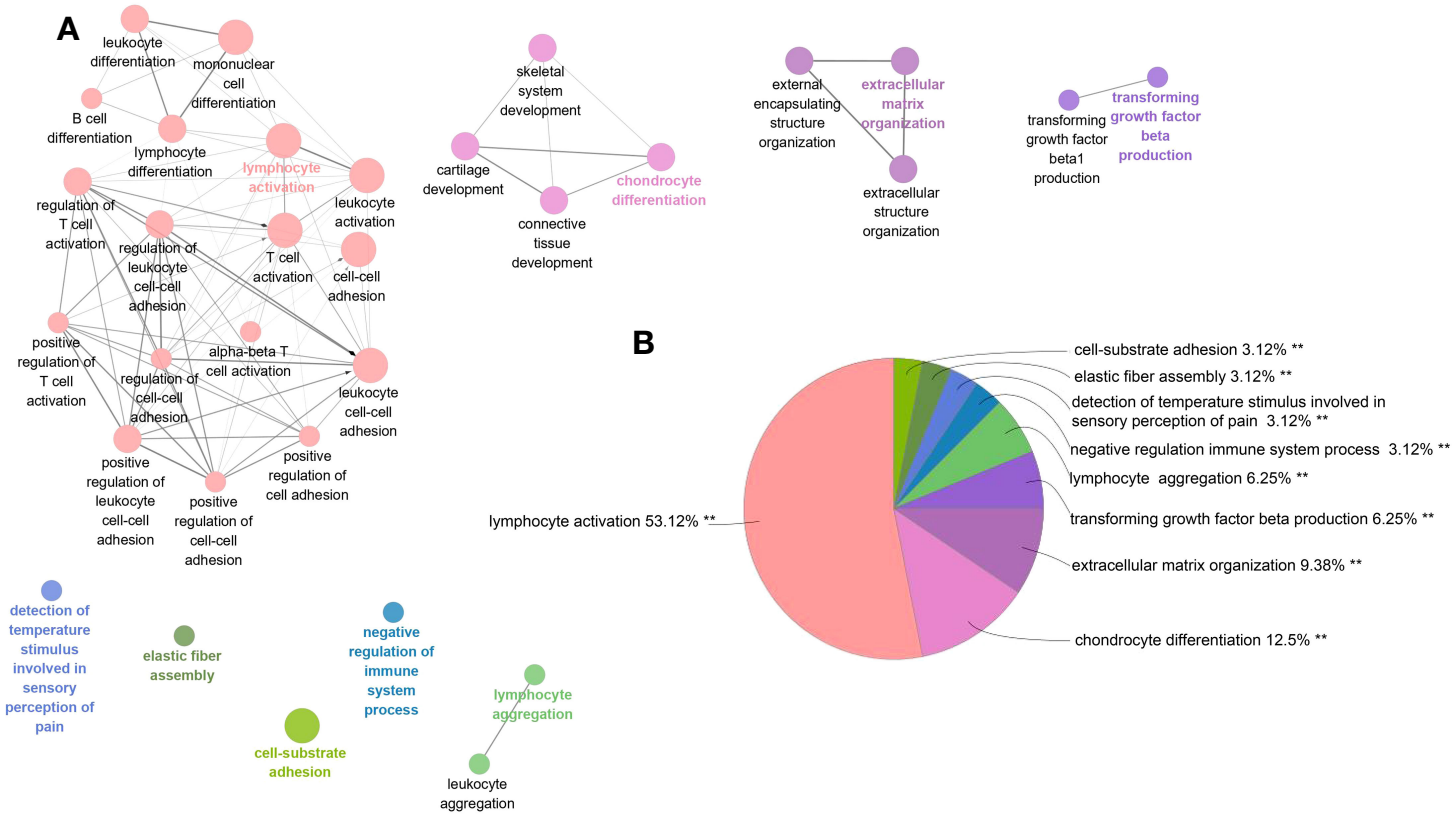
ClueGO enrichment analysis of dataset 1. **(A)** The interaction network of GO terms generated by the Cytoscape plug-in ClueGO. The significant term of each group is highlighted. **(B)** Proportion of each GO terms group in the total. GO, gene ontology. **p < 0.05.

**Figure 3:**
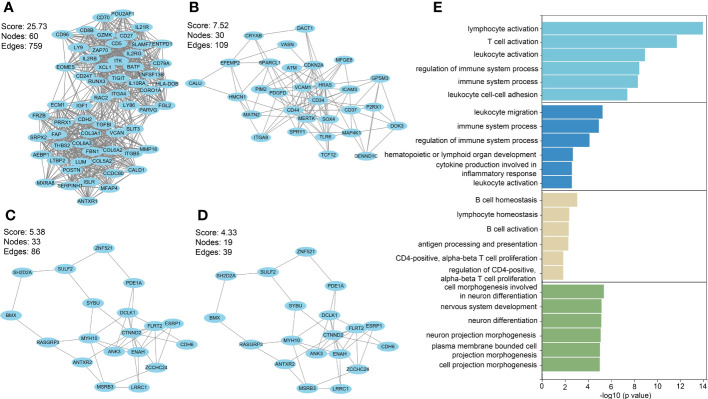
**(A–D)** PPI network analysis was performed on gene set 1, and four clusters were identified. **(E)** GO analysis was performed on each cluster.

Among PF and PH, 313 genes in the most relevant positive correlation module overlapped and were defined as gene set 1 (**Figure 1E**). To explore the potential function of gene set 1, Gene Ontology (GO) enrichment was analyzed using ClueGO. The first significantly enriched GO terms for Biological Process (BP) are “lymphocyte activation,” which accounted for 53.12% of the total GO terms (**Figures 2A, B** ), confirming that this pathway may be essential in both PF and PH. Then, we constructed a protein-level PPI network for dataset 1 (**Figures 3A-D**). Four clusters were extracted using MCODE analysis, and cluster 1 contains 60 nodes and 759 edges (score = 25.73). We performed functional enrichment analysis on the genes of the four clusters respectively and selected the first 10 immune-related pathways (**Figure 3E**). GO enrichment analysis showed that the genes in cluster 1 were mainly related to the lymphocyte activation pathway; this was consistent with the enrichment of dataset 1. Therefore, this gene cluster belongs to the gene part shared by PF in PH.

### 3.4 DEGs in PF and PH

**Figure 4:**
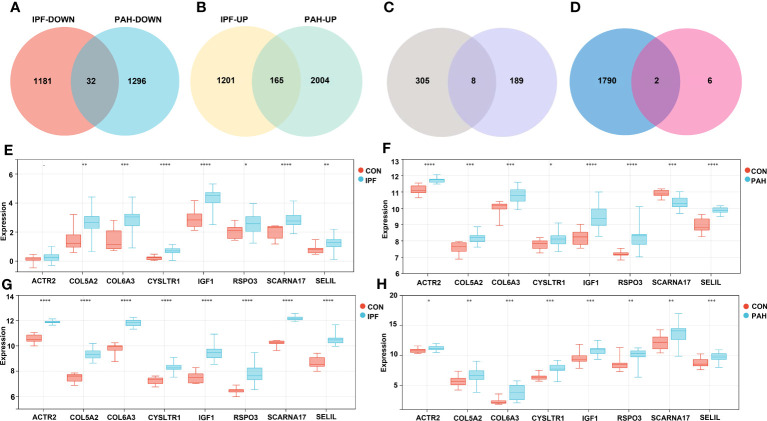
Validation Group: **(A, B)** Differential gene analysis of PF dataset GSE110147 and PH dataset GSE15197, respectively: downregulated intersection gene and upregulated intersection gene. **(C)** The intersection gene set 1 and gene set 2 were selected, and there were eight key genes. **(D)** The intersection of immune-related genes with the eight key genes was retrieved in the ImmPort database. Analysis of expression levels of eight key genes in the verification group of the discovery group: GSE53845 **(E)**, GSE113439 **(F)**, GSE110147 **(G)**, and GSE15197 **(H)**. IPF, idiopathic pulmonary fibrosis; PAH, pulmonary arterial hypertension; CON, control. **p < 0.01; ***p < 0.001; ****p < 0.0001.

We performed DEG analyses of GSE110147 and GSE15197 to validate our results. Among the 2,579 DEGs in GSE110147, 1,213 were significantly downregulated and 1,366 were significantly upregulated. In GSE15197, 3,497 DEGs were identified, including 1,328 downregulated and 2,169 upregulated genes. Further cross-analysis of GSE110147 and GSE15197 revealed that there were 32 co-downregulated genes and 165 co-upregulated genes in PF and PH, defined as gene set 2 and expressed by Venn diagram (**Figures 4A, B**). There were eight overlapped genes, *ACTR2, COL5A2, COL6A3, CYSLTR1, IGF1, RSPO3, SCARNA17* and *SEL1L*, in GS1 and GS2 (**Figure 4C**). Notably, *COL5A2, IGF1*, and *COL6A3* are contained in cluster 1, and *RSPO3* is contained in cluster 3. At the same time, the expression level of the overlapped genes in the four datasets was visualized (**Figures 4E-H**).

### 3.5 Immune-related common genes and immune infiltration analysis in PF and PH

**Figure 5:**
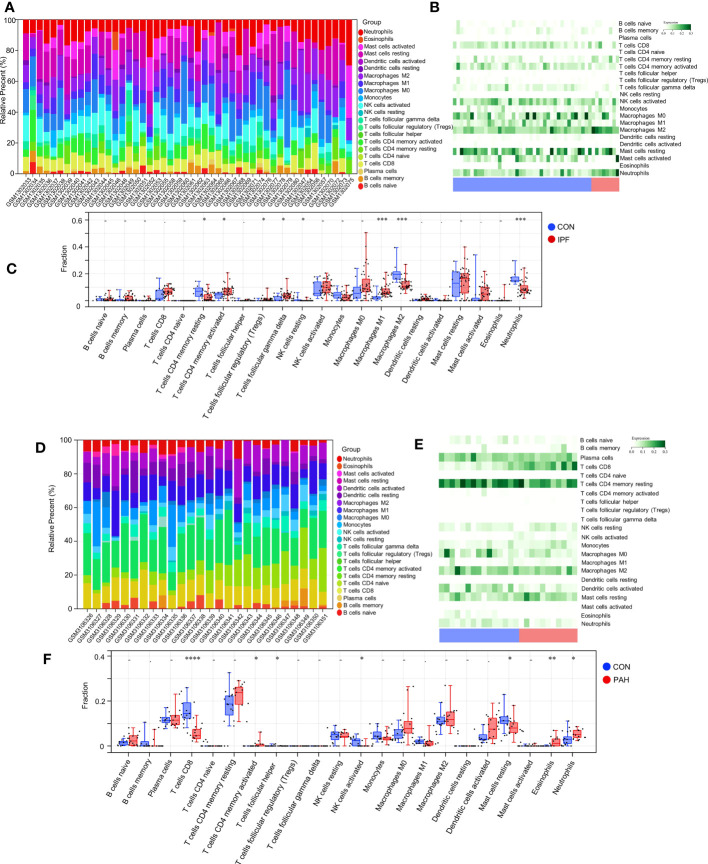
Immune infiltrates between PF and normal controls: **(A)** Relative percentage of 22 immune cells; **(B)** Heat map of 22 immune cells; **(C)** Difference in immune infiltrates between PH and normal controls. Immune infiltrates between PH and normal controls: **(D)** Relative percentages of 22 immune cells; **(E)** Heat maps of 22 immune cells; **(F)** Differences in immune infiltrates between PH and normal controls. IPF, idiopathic pulmonary fibrosis; PAH, pulmonary arterial hypertension; CON, control. *p < 0.05; **p < 0.01; ***p < 0.001; ****p < 0.0001.

**Figure 6:**
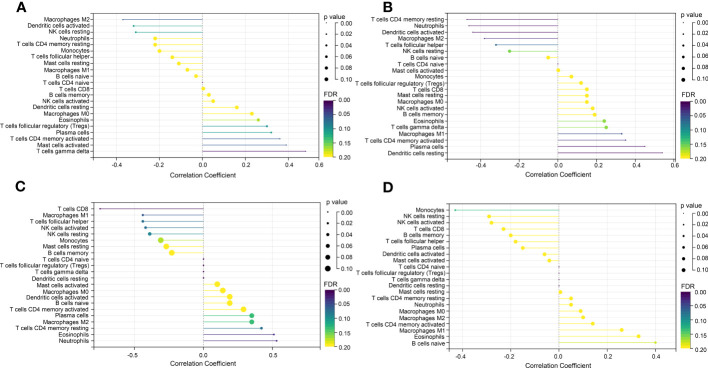
Correlation between *IGF1* and *CYSLTR1* and immune cells. **(A)** Correlation between *IGF1* and immune cells (in GSE53845). **(B)** Correlation between *CYSLTR1* and immune cells (in GSE53845). **(C)** Correlation between *IGF1* and immune cells (in GSE113439). **(D)** Correlation between *CYSLTR1* and immune cells (in GSE113439). The length of the line represents the correlation between genetic biomarkers and immune cells. The size and color of the points represent p-values and FDR, respectively, and the smaller the point, the greater the difference.

In the enrichment assay, immune-related biological functions were significantly enriched in PF and PH. Therefore, we further performed CIBERSORT to predict immune cell infiltration between patients with the disease and control groups. The percentage of each of the 22 immune cells in each sample was shown in the bar and heat graphs of GSE53845 (**Figures 5A, B**) and GSE113439 (**Figures 5D, E**). The box diagram of the differences in immunocyte infiltration when samples were classified into disease and control groups showed significant differences of T cells CD4 memory resting, T cells CD4 memory activated, T cells regulatory (Tregs), T cells gamma delta, natural killer (NK) cells resting, Macrophages M1, Macrophages M2, and Neutrophils in PF patients compared with those in controls (**Figure 5C**). Compared with the control group, there were significant differences in T cells CD8, T cells CD4 memory activated, T cells follicular helper, NK cell activated, Mast cells resting, Eosinophils, and Neutrophils in patients with PH (**Figure 5F**). As can be seen from the results, T cells CD4 memory activated was a common significantly different immune feature to PF and PH. In addition, *IGF1* and *CYSLTR1* were the shared genes between 1,792 immune-related genes retrieved from the ImmPort database and eight common genes (**Figure 4D**). *IGF1* and *CYSLTR1* were also related to several immune cells (**Figure 6**).

### 3.6 Demonstration of the expression of overlapped genes in PF-PH patients

**Figure 7:**
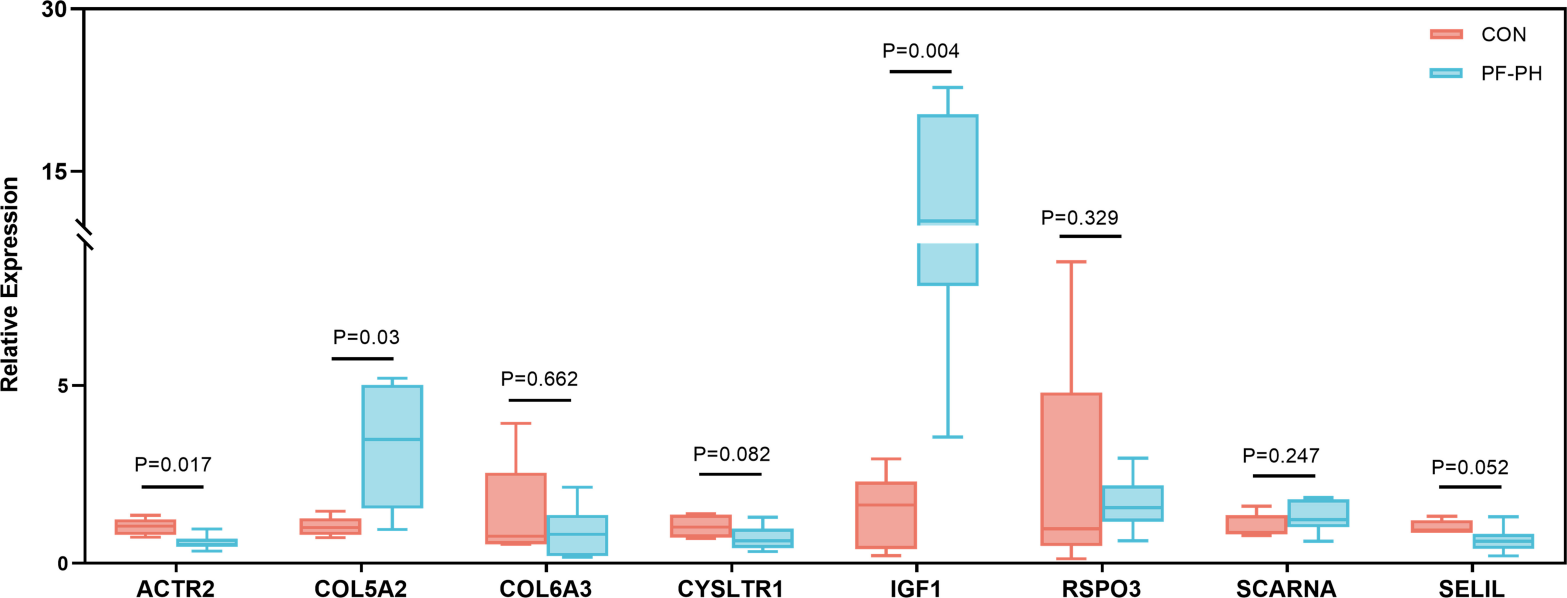
RT-PCR for the expression of common genes of PF and PH in PF-PH patients and healthy donors. PF, pulmonary fibrosis; PH, pulmonary hypertension; CON, control.

To further demonstrate the expression of overlapping genes in PF-PH patients, we performed RT-PCR in six lung tissue samples from PF-PH patients and five healthy donors. The results showed that *IGF1* and *COL5A2* expression was distinctly increased in PF-PH samples compared with healthy samples (**Figure 7**), which is consistent with previous findings in DEGs of PF and PH.

### 3.7 Effect of siRNA *IGF1* on cell function

**Figure 8:**
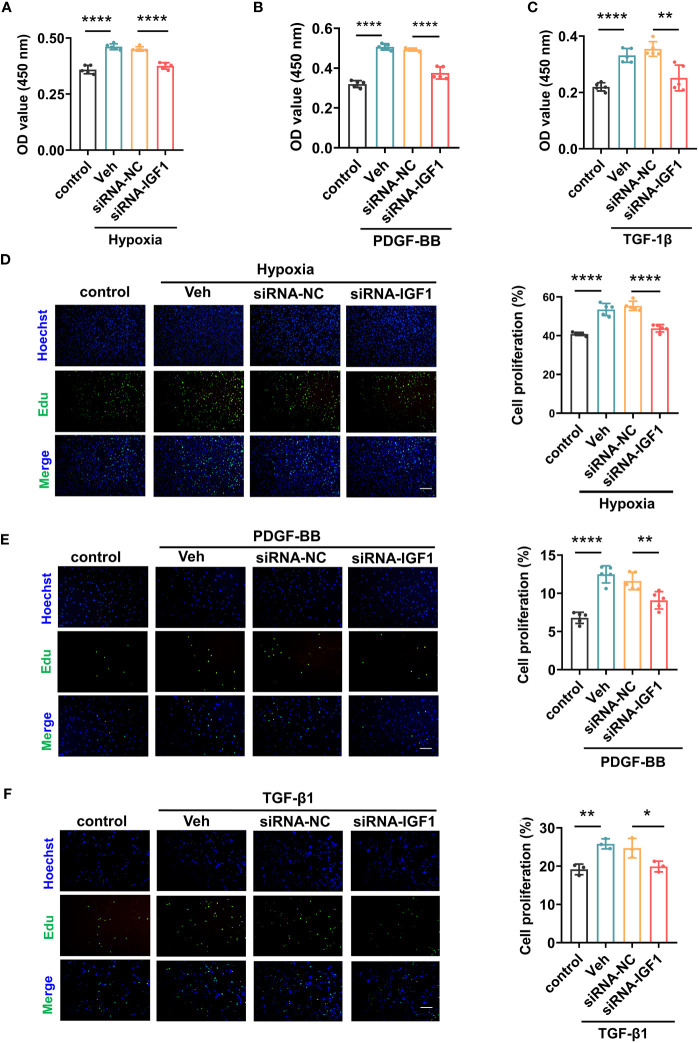
Effects of *IGF1* on the proliferation of PMECs, PASMCs, and fibroblasts. The CCK-8 Kit test proliferation of PMECs **(A)**, PASMCs **(B)**, and fibroblasts **(C)** with siRNA *IGF1*. The EdU Kit test proliferation and analysis results of PMECs **(D)**, PASMCs **(E)**, and fibroblasts **(F)**. All data are presented as the mean ± SEM. Scale bar 100 μm. *p < 0.05; **p < 0.01; ****p < 0.0001. Veh, vehicle; PDGF-BB, platelet-derived growth factor-BB; TGF-β1, transforming growth factor-β1.

**Figure 9:**
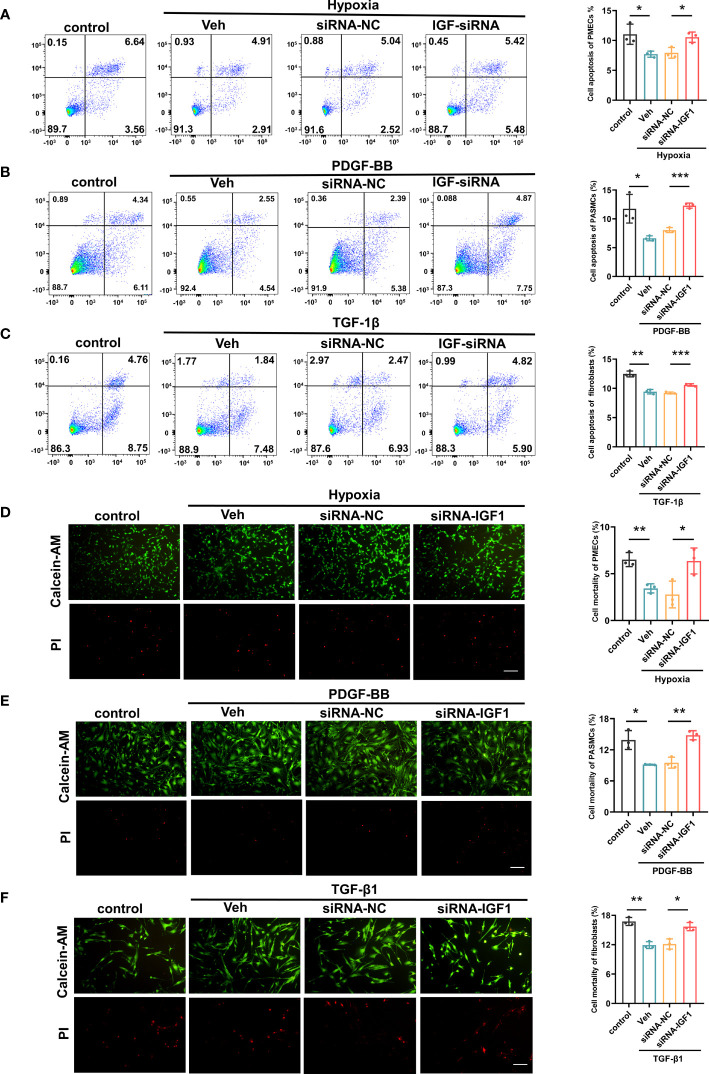
Effects of *IGF1* on the apoptosis and mortality of PMECs, PASMCs, and fibroblasts. The flow cytometry test apoptosis of PMECs **(A)**, PASMCs **(B)**, and fibroblasts **(C)** with siRNA *IGF1*. The AM/PI Kit test mortality and analysis results of PMECs **(D)**, PASMCs **(E)**, and fibroblasts **(F)**. All data are presented as the mean ± SEM. Scale bar 100 μm. *p < 0.05; **p < 0.01; ***p < 0.001. Veh, vehicle; PDGF-BB, platelet-derived growth factor-BB; TGF-β1, transforming growth factor-β1.

In view of the upregulation of *IGF1* in lung tissue of patients with PF-PH, we used siRNA *IGF1* to reduce the expression of IGF in PMECs, PASMCs, and fibroblasts to further study the effect of *IGF1* on cell function. Hypoxia, PDGF-BB, and TGF-β1 were used to induce the proliferation of PMECs, PASMCs, and fibroblasts, respectively. As shown in **Figures 8A–C**, the cell proliferation was increased obviously after stimulation and significantly inhibited after adding siRNA *IGF1*. In addition, we used EdU Kit to verify the cell proliferation, and the results were consistent (**Figures 8D–F**). Then, using flow cytometry and AM/PI Kit, we identified that siRNA *IGF1* could reduce the apoptosis rate and death rate promoted by hypoxia, PDGF-BB, and TGF-β1 in PMECs (**Figures 9A, D**), PASMCs (**Figures 9B, E**), and fibroblasts (**Figures 9C, F**), respectively.

### 3.8 The ability of IGF1 to target drugs

**Figure 10:**
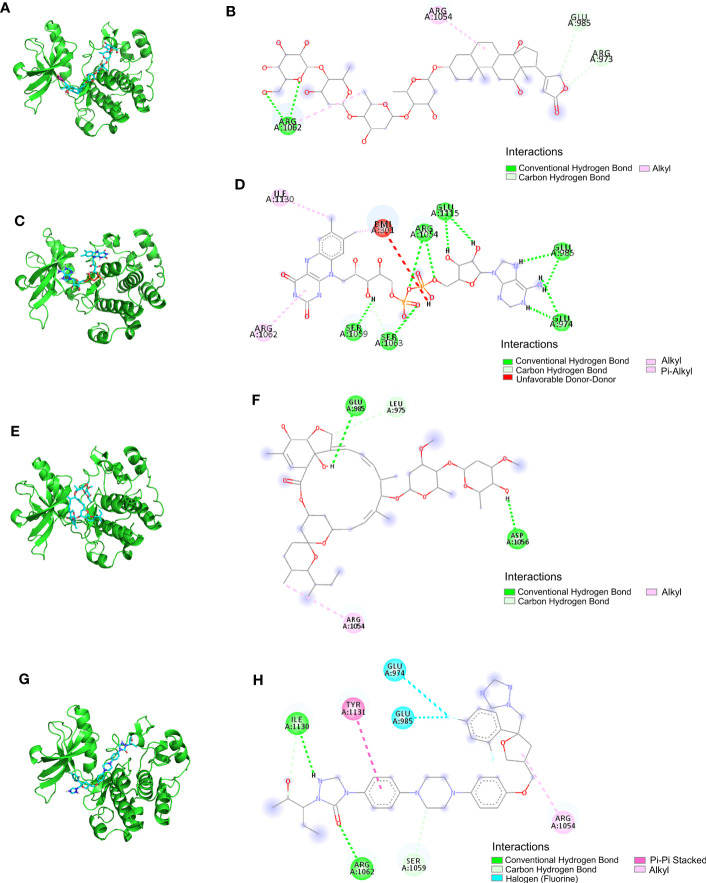
The ability of IGF1 to target deslanoside **(A, B)**, flavitan **(C, D)**, ivermectin **(E, F)**, and posaconazole **(G, H)**.

The LibDock docking results showed that 2,720 compounds in 7,929 were successfully docked to protein IGF1. The higher the LibDock score, the closer the interaction between molecules and proteins. We selected the top 20 compounds of LibDock score (**Table 1**) to enter the next research. Subsequently, the lower the binding ability, the more stable the binding between the ligand and the receptor (the lower the binding energy, the better the binding) in vina docking results. Therefore, among the first 20 compounds, we screened out the compounds with binding energy less than -7.0 kcal/mol by vina (22), namely, deslanoside (**Figure 10A**), flavitan (**Figure 10C**), ivermectin (**Figure 10E**), and posaconazole (**Figure 10G**), which have strong binding activity with IGF1. The results also show the interaction between these drugs and IGF1. Deslanoside forms hydrogen bonds (HBs) with ARG1062, GLU985, and ARG973 of IGF1 and forms alkyl bonds with ARG1062 and ARG1054 (**Figure 10B**). Six HBs were formed between flavitan and IGF1, namely, SER1059, SER1063, ARG1054, GLU1115, GLU985, and GLU974, and also formed alkyl with ILE1130 and pi-alkyl with ARG1062 (**Figure 10D**). Ivermectin forms HBs with GLU985 and ASP1056 of IGF1 and alkyl with ARG1054 (**Figure 10F**). Posaconazole forms HBs with ILE1136, ARG1062, and SER1059 of IGF1, alkyl with ARG1054, pi-pi stacked with TYR1131, and halogen with GLU974 and GLU985 (**Figure 10H**).

**Table 1:** The top 20 compounds of LibDock score.

Index	Name	LibDockScore
1	Ritonavir	203.982
2	Octreotide	199.237
3	Saralasin	198.04
4	Pralmorelin hydrochloride`	196.064
5	Proglumetacin	195.161
6	Polymyxin B1	188.494
7	Amogastrin	186.031
8	Bimosiamose disodium	184.755
9	Cargutocin	184.44
10	Argipressin	183.96
11	Ivermectin	183.392
12	Desmopressin	182.221
13	Pentagastrin	181.88
14	Flavitan	181.749
15	Colistin	180.815
16	Argiprestocin	178.903
17	Indinavir	177.897
18	Tetragastrin	177.388
19	Deslanoside	176.341
20	Posaconazole	175.391

## 4 Discussion

The recent approval of the first PAH therapy for treating PF-associated PH represents an encouraging advancement, which indicates that despite the heterogeneity in patients, shared molecular mechanisms contribute to the perpetuation of the fibrotic process and pulmonary vascular remodeling (23). In this study, we conducted WGCNA to find clusters (modules) of highly relevant genes, explored the relationship between gene networks and the two diseases (17), and then obtained the DEGs, which refer to the specific genes expressed between healthy subjects and patients with PF or pulmonary vascular remodeling. After integration, the final “important” intersecting genes were discovered, probably the potential vital genes and therapeutic targets of PF-associated PH.

Drawing support from WGCNA, we first described several intersection genes of PF and pulmonary vascular remodeling, most of which are enriched in lymphocyte activation. Then, we performed CIBERSORT analytical tool (24) to specifically analyze the essential fractions of 22 subpopulations of immune cells and observed that activated memory CD4+ T cells were upregulated in the lung of both PF and PH patients compared with control. Further evaluation and validation allowed us to identify *IGF1* as a potential immune-related critical gene for pulmonary vascular remodeling and fibrosis.

Immunity is one of the critical factors involved in the pathogenesis of PH and PF besides genetic predisposition (25, 26), epigenetic regulation (27, 28), metabolic derangement (26), and environment insults (28, 29). Both innate immunity and adaptive immunity contribute to all forms of PF and pulmonary artery vascular remodeling. Pulmonary vasculopathy in patients with PH is characterized by varying degrees of perivascular inflammatory infiltration, including T lymphocytes, B lymphocytes, macrophages, dendritic cells, and mast cells (30). Meanwhile, those immune cells are also important players in fibroblast biology and fibrogenesis (31, 32). In our study, we first demonstrated that the shared genes between PF and PH are mainly functionally enriched in lymphocyte activation, which refers to a series of cellular biochemical changes and processes of lymphocytes under the stimulation of antigen or mitogen, including the transmission of the cell activation signal and the activation of a variety of related enzymes. According to their migration, surface molecules, and functions, they can be divided into T lymphocytes (also known as T cells), B lymphocytes (also known as B cells), and NK cells.

Using CIBERSORT, we predicted that CD4+ T cell activation was a common immune feature to PF and PH. CD4+ T cells can differentiate into several effector subsets, such as Th1, Th2, Th17, Tregs, and Tr1 cells, depending on factors in the local environment, such as cytokines and T cell receptor (TCR) signaling strength (33). Previous studies have already described a specific depletion of CD4+ T cells in peripheral venous blood of patients with PH (34) and PF (35); this significant downregulation is associated with poor outcomes in patients with PF (35) and PH (36). While in the perivascular (37) and fibrosis area (38), a significantly increased helper T cell (CD4) prevalence could be found, such as Th17, which produces several cytokines promoting pulmonary vascular remodeling (36) and fibrogenesis (38). Unlike Th17, Treg cells, especially their CD4+/CD25+/Foxp3+ phenotype, are essential for maintaining immune system homeostasis, especially in long-term inflammatory responses. They inhibit the activity of other T cells, especially CD8+ cytotoxic T cells, limit the immune response (39), reduce endothelial injury, and then impede PH-mediated vascular remodeling (40, 41) by upregulating COX-2, PTGIS, HO-1, and PD-L1 in the media layer of the pulmonary arteriole (40). A previous study identified Treg-associated genes in the progression of PH, and the gene signature based on these genes was revealed to be a novel indicator to distinguish PH from controls (42). The imbalanced Th1/Th2 immune response has been considered central to the pathogenesis of PF (43); Treg impairment is also dedicated to the development of PF (44). Global impairment of CD4+CD25+FOXP3+ Tregs has been identified in the peripheral blood and bronchoalveolar lavage fluid in IPF patients and is strongly correlated with disease severity (45). Tregs can attenuate fiber recruitment and PF by inhibiting the CXC chemokine ligand (CXCL)12 (46). Recently, Ichikawa et al. (47) found that depletion of CD69hiCD103hiFoxp3+ Tregs resulted in substantially higher levels of lung fibrosis in mice during chronic exposure to *Aspergillus fumigatus* because tissue-resident CD44hiCD69hi CD4+ T cells had increased expression of fibrosis-related genes. Nevertheless, a profibrosis effect of CD4+CD25hiFoxp3+ Tregs has been shown in bleomycin-induced PF (48) and other types of fibrosis animals (31), so the potential role of Tregs in PF remains uncertain and can be controversial.

To further identify the hub gene, we validated 313 shared genes of PF and PH in two GEO datasheets by differential gene expression analysis. Then, we confirmed their mRNA expression in the lung tissues of PF-PH patients. Ultimately, the *IGF1* gene with high functional significance was selected as a central shared gene related to immunity in PF and PH. It is a single-chain polypeptide with a high sequence homology to pro-insulin and has been reported as a critical factor for developing T and B cells from pluripotent precursors (49). IGF1 interacts with the IGF1 receptor, whose signaling regulates T-cell proliferation, apoptosis, and Treg cell function by inducing phosphorylation of phosphoinositide 3-kinase/Akt pathway (50, 51). Meanwhile, *IGF1* is also highly expressed in lung fibroblasts (52, 53), pulmonary artery endothelial cells, and PASMCs (54). *In vivo*, IGF1 can increase the levels of phosphorylated AKT at residue position S473 in PASMCs and elevate the expression of endothelin-1 (ET-1) in pulmonary artery endothelial cells (54). In neonatal mice, hypoxia exposure can modulate DNA methylation in the *IGF1* promoter region, upregulating its levels in the lungs (54). Smooth muscle cell (SMC)-specific deletion of *IGF1* reduced the proliferation of PASMCs and attenuated hypoxia-induced pulmonary vascular remodeling, right ventricular hypertrophy, and right ventricular systolic pressure (55). Consistent with our analysis, *IGF1* has been shown to be overexpressed in fibrotic fibroblasts, especially class 1 and IGF-1Ea variants, and promoting fibroblast proliferation and extracellular matrix deposition (53).

Blocking the IGF1 pathway by a monoclonal antibody against the IGF1 receptor exhibited a protective effect on bleomycin-induced lung injury in animal models (56). All of these indicate that IGF1 was probably involved in the pathogenesis of fibrogenesis and PH. As IGF1 has long been implicated in various tumorigeneses, including pancreatic cancer, breast cancer, Ewing sarcoma, and melanoma, several small molecules and monoclonal antibodies targeting IGF1 signaling have been developed (57, 58). Further translational research is needed to provide direct evidence proving that inhibition of IGF1 could become a potential new therapeutic target for PF and PH.

Among the eight shared genes we discovered, *COL5A2* is another gene that has been successfully validated. Studies have shown that *COL5A2* is related to fibrosis. Foxf2 interacted with Smad6, downregulated *COL5A2* transcription, and reduced fibrosis (59). Moreover, C57BL/6 mice presented with increased tissue elastance and a nonspecific interstitial pneumonia histologic pattern in the lung, combined with the thickening of the small and medium intrapulmonary arteries, increased fibrosis, and increased *COL5A2* gene expression (60). In addition, *COL5A2* expression linked to cell morphogenesis, angiogenesis, and blood vessel development (61). Gene co-expression network revealed that *COL5A2* shows predictive potential in myocardial infarction, which may be a new candidate marker for identifying and treating ischemic cardiovascular diseases (62).

The results of molecular docking show that four important compounds (deslanoside, flavitan, ivermectin, and posaconazole) have strong binding activity with the target protein IGF1, and the main forms of interaction between components and targets are HBs, alkyl interaction, π-π stacking, and halogen bonding. They make that compound have a strong binding force with the target protein IGF1.

Deslanoside is widely used to treat a variety of heart diseases, such as arrhythmia and hypotension, mainly by inhibiting Na/K-ATPase. Recently, *in vitro* and *in vivo* studies have shown that deslanoside reduces the proliferation rate of tumor cells, causes G2/M cell cycle arrest, induces cell apoptosis, reduces colony formation, and inhibits migration and invasion, thus exerting anticancer activity (63). Flavin is an important component of flavin adenine dinucleotide (FAD) and riboflavin. FAD was effective in recycling glutathione disulfide *in vivo* and promoted alveologenesis but did not impact alveolar fluid clearance nor attenuate fibrosis following high fraction inspired oxygen exposure (64). However, ATP content was increased, and the content of free fatty acids and reactive oxygen species were decreased by FAD *in vivo* and *in vitro* (65). FAD can inhibit pathological cardiac hypertrophy and fibrosis through activating short-chain acyl-CoA dehydrogenase, thus preventing them from developing into heart failure (65). Riboflavin also works as an antioxidant by scavenging free radicals. Administration of lipopolysaccharide (LPS) resulted in marked cellular changes including interstitial edema, hemorrhage, infiltration of PMNs, which were reversed by riboflavin administration (66). Ivermectin is one of the most important drugs for the control of parasitic infection, which has antiviral activity against many DNA and RNA viruses, including coronavirus disease-2019 (COVID-19) (67). With regard to its anti-inflammatory properties, ivermectin has been proven to significantly reduce the recruitment of immune cells and the production of cytokines (68) and inhibit TNF-α and IL-6 induced by LPS (69). Posaconazole is being used for invasive pulmonary aspergillosis at present (70). In addition, posaconazole accumulates in lung tissue, especially in macrophages (71). This characteristic allows for the long duration of action commonly observed in epithelial cells (71).

We acknowledge that this study has some limitations. First, the datasets used were all derived from transcriptomics. The use, mutual verification, and supplement of other omics must be addressed. Further analysis and verification of other omics will be needed after this research. Second, the sample size in RT-PCR validation was small because obtaining lung samples from patients and controls is challenging. Third, heterogeneity of PF and PH has not been considered in this study. Fourth, further demonstration of machinery by future studies is necessary.

In conclusion, we proposed that lymphocyte activation might be a vital pathway affecting the pathogenesis of PF and PH and identified *IGF1* as the critical immune-related common gene. These data findings provide a basis for future research and new potential therapeutic targets for the future treatment of PF and PH.

## Data availability statement

The original contributions presented in the study are included in the article/**Supplementary Material**. Further inquiries can be directed to the corresponding authors.

## Ethics statement

The studies involving humans were approved by the Ethics Committee of Shanghai Pulmonary Hospital (numbers: K22-137Y). The studies were conducted in accordance with the local legislation and institutional requirements. The participants provided their written informed consent to participate in this study.

## Author contributions

HZ, LW, and YY investigated the literature research, got the data, and analyzed the data. Q-HZ and JH wrote the article. RJ and C-JL modified the figures. H-LQ and Y-QM revised the article. W-HW, PY, and S-GG conceived the idea of the study, designed the steps of the study, and directed the data analysis. All authors contributed to the article and approved the submitted version.
