# Supplementary material for: Identification of the shared gene signatures between pulmonary fibrosis and pulmonary hypertension using bioinformatics analysis
Source: Front Immunol. 2023 Sep 4;14:1197752. doi: 10.3389/fimmu.2023.1197752 (PMC10507338; doi:10.3389/fimmu.2023.1197752)
Supplement: Supplementary file 1 [file Table_1.docx]

Supplement Table 1. Primers for the differentially expressed mRNAs used in RT-PCR

| Analysis name | GEO | Packages |
| --- | --- | --- |
| Probe name conversion gene name | GSE53845 | GEOquery  dplyr |
|  | GSE113439 | GEOquery  hugene10sttranscriptcluster.db |
| WGCNA | GSE53845 | WGCNA |
|  | GSE113439 | WGCNA |
| GO | GSE53845 | org.Hs.eg.db |
|  | GSE113439 | org.Hs.eg.db |

GEO: Gene Expression Omnibus; WGCNA: Weighted Correlation Network Analysis; GO: Gene Ontology.
