# Supplementary material for: Identification of the shared gene signatures between pulmonary fibrosis and pulmonary hypertension using bioinformatics analysis
Source: Front Immunol. 2023 Sep 4;14:1197752. doi: 10.3389/fimmu.2023.1197752 (PMC10507338; doi:10.3389/fimmu.2023.1197752)
Supplement: Supplementary file 2 [file Table_2.docx]

Supplement Table 2. Primers for the differentially expressed mRNAs used in RT-PCR

| Gene | Species |  | Primer sequence |
| --- | --- | --- | --- |
| ACTR2 | Human | Forward | ggatacgccttcaaccactc |
|  |  | Reverse | gccagtttctgctcttgctc |
| COL5A2 | Human | Forward | atttcagcaaacccatccag |
|  |  | Reverse | aagcgaactgagaccctctg |
| COL6A3 | Human | Forward | cgctttgcagatcaataaca |
|  |  | Reverse | ttgcagatgtccaagcaaac |
| CYSLTR1 | Human | Forward | tgcgtgggcttcctctaata |
|  |  | Reverse | ctgctttgtgcctctttctg |
| IGF1 | Human | Forward | ccggagctgtgatctaagga |
|  |  | Reverse | gatgggggctgatacttctg |
| RSPO3 | Human | Forward | acaccttggaaagtgccttg |
|  |  | Reverse | tttttcccttcttcgtgcat |
| SCARNA | Human | Forward | ggggtgcggtgattgtagta |
|  |  | Reverse | cgccacgttttcataatcct |
| SELIL | Human | Forward | tgaagtggcacaaagcaatg |
|  |  | Reverse | gccctgttccaatgtagcaa |
| β-actin | Human | Forward | caccattggcaatgagcggttc |
|  |  | Reverse | aggtctttgcggatgtccacgt |
