# Supplementary material for: Identification of the shared gene signatures between pulmonary fibrosis and pulmonary hypertension using bioinformatics analysis
Source: Front Immunol. 2023 Sep 4;14:1197752. doi: 10.3389/fimmu.2023.1197752 (PMC10507338; doi:10.3389/fimmu.2023.1197752)
Supplement: Supplementary file 3 [file Table_3.docx]

Supplement Table 3 GSE53845 and GSE113439 subject characteristics

| GSE53845[1] | | GSE113439[2] | |
| --- | --- | --- | --- |
| Variable | N=40 | Variable | N=15 |
| Age, years | 60.6 ± 8.8 | Age (years) | 41 ± 12 |
| Male gender, N (%) | 33 ± 83 | Gender (male/female) | 3/12 |
| VATS biopsy, Explant, N | 11, 29 | BMI (kg/m^2^) | 23 ± 4 |
| Ever smoked % | 68 | RVSP (mm Hg) | 58 ± 19 |
| FVC, % predicted | 55.8 ± 17.2 | DLco, % predicted | 57 ± 26 |
| TLC, % predicted | 55.7 ± 16.2 | 6-Min walk distance (m) | 271 ± 122 |
| DLCO, % predicted | 34.1 ± 13.8 | mPAP (mmHg) | 56 ± 12 |
|  |  | PVR (dyn/s/cm^5^) | 1197 ± 595 |
|  |  | Pre-transplant therapy |  |
|  |  | Bosentan | 6, 40% |
|  |  | Sildenafil | 4, 27% |
|  |  | Epoprostenol | 12, 80% |
|  |  | Dual therapy | 10,67% |

Values are reported as mean ± standard deviation.

Abbreviations: VATS, video assisted thoracoscopic biopsy; FVC, forced vital capacity; TLC, total lung capacity; DLCO, diffusion capacity of carbon monoxide; BMI, body mass index; DLCO, diffusing lung capacity for carbon monoxide; mPAP, mean pulmonary arterial pressure; PVR, pulmonary vascular resistance; RVSP, right ventricle systolic pressure (2D echocardiogram).

**References**

[1] D.J. DePianto, S. Chandriani, A.R. Abbas, G. Jia, E.N. N'Diaye, P. Caplazi, S.E. Kauder, S. Biswas, S.K. Karnik, C. Ha, Z. Modrusan, M.A. Matthay, J. Kukreja, H.R. Collard, J.G. Egen, P.J. Wolters, and J.R. Arron, Heterogeneous gene expression signatures correspond to distinct lung pathologies and biomarkers of disease severity in idiopathic pulmonary fibrosis. Thorax 70 (2015) 48-56.

[2] M. Mura, M.J. Cecchini, M. Joseph, and J.T. Granton, Osteopontin lung gene expression is a marker of disease severity in pulmonary arterial hypertension. Respirology 24 (2019) 1104-1110.
